# Influencing Factors Associated With Infantile Hemangioma: A Case–Control Study Among Chinese Pediatric Populations

**DOI:** 10.1111/1346-8138.70446

**Published:** 2026-08-24

**Authors:** Xinyu Liu, Dan Song, Liang Wang, Changfeng Wang, Lei Guo

**Affiliations:** ^1^ Department of Vascular Anomalies and Interventional Radiology Children's Hospital Affiliated to Shandong University Jinan China; ^2^ Department of Vascular Anomalies and Interventional Radiology Jinan Children's Hospital Jinan China

**Keywords:** case–control study, Chinese population, epidemiology, infantile hemangioma, risk factors

## Abstract

Infantile hemangioma (IH) is the most common benign vascular tumor of infancy. While its epidemiology is well‐documented in Caucasian populations, large‐scale systematic studies regarding clinical characteristics and risk factors in the Chinese population remain limited. This study aims to elucidate the clinical features of IH in Chinese children and investigate risk factors associated with its development. A retrospective case–control study was conducted at the Children's Hospital of Shandong University involving children under 3 years of age treated between January and December 2024, including 1 841 children with IH (case group) and 1 894 contemporaneous non‐IH patients (control group). Clinical phenotypes were analyzed, and potential risk factors were screened using univariate analysis followed by multivariate logistic regression to identify independent determinants. The cohort exhibited a female predominance (male‐to‐female ratio 1:1.88), with the head and neck being the most common lesion site (46.7%). Multivariate analysis identified multiple gestation (*OR* = 4.50, 95% *CI*: 3.55–5.70) as the strongest independent risk factor, followed by female gender (*OR* = 2.72), progestogen therapy (*OR* = 2.25), low birth weight (*OR* = 2.11), preterm birth (*OR* = 1.65), and pregnancy‐related diseases (*OR* = 1.57). Notably, breastfeeding was identified as an independent protective factor against IH (*OR* = 0.76, 95% *CI*: 0.58–0.99), whereas caesarean section was not significantly associated with IH risk. This study confirms that IH pathogenesis in the Chinese population is multifactorial, strongly associated with female gender, intrauterine hypoxia (multiple gestation, pregnancy‐related diseases), and perinatal hormone exposure. The protective effect of breastfeeding suggests a potential avenue for risk mitigation, and these findings provide epidemiological evidence to support early risk stratification and surveillance strategies for high‐risk infants.

## 1 Introduction

Infantile hemangioma (IH) is the most common benign vascular tumor in infancy and early childhood, arising primarily from abnormal proliferation of vascular endothelial cells during the embryonic period [1]. Although most IHs follow a self‐limiting course, approximately 70% of untreated children develop sequelae such as telangiectasia and excessive accumulation of fibrofatty tissue [1]. Furthermore, rapidly growing lesions or those located in critical anatomical sites may cause ulceration, airway obstruction, or permanent functional impairment [2], imposing significant psychological and financial burdens on children and their families. The diagnosis and categorization of these lesions in modern clinical practice are primarily guided by the updated International Society for the Study of Vascular Anomalies (ISSVA) classification system, which serves as the diagnostic gold standard [3].

Epidemiological data, including a recent comprehensive global meta‐analysis, indicate a global prevalence of IH of approximately 2.8%, with marked racial disparities: Caucasians exhibit a higher risk than Asian and African populations [2]. Several risk factors associated with the occurrence of IH have been identified, most of which relate to the perinatal period and maternal conditions. Multiple epidemiological studies have confirmed that Caucasian ethnicity, preterm birth, low birth weight, multiple gestation, female sex, progesterone therapy, and family history constitute strong independent risk factors for IH [4, 5, 6, 7]. Additional reported maternal factors include placental abnormalities, pre‐eclampsia, maternalanemia, and the use of progestogen medications during pregnancy [8, 9].

However, this geographical and racial heterogeneity suggests complex interactions between genetic and environmental factors in IH pathogenesis. Variations in genetic background, environmental exposures, and lifestyle habits across different populations may lead to distinct epidemiological characteristics and risk profiles. While the clinical features and risk factors of IH have been extensively documented in Caucasian cohorts, systematic data regarding the Chinese population remain relatively scarce [10, 11]. Existing domestic studies are often limited by smaller sample sizes, carrying a potential risk of bias and yielding inconsistent conclusions. Therefore, to comprehensively elucidate the clinical characteristics and associated risk factors of IH within the Chinese demographic, this study enrolled a large‐scale cohort. By systematically analyzing these clinical phenotypes, we aim to provide robust, population‐specific evidence to support early screening and targeted interventions for IH.

## 2 Methods

### 2.1 Study Design and Ethical Considerations

This study employs a case–control design to investigate factors influencing the incidence of IH. Participants comprised children under 3 years of age receiving treatment at the Department of Hemangioma and Interventional Vascular Surgery, Children's Hospital of Shandong University, between January and December 2024.

This study strictly adheres to the principles of the Declaration of Helsinki and has received approval from the Research Ethics Committee (Approval No. SDFE‐IRB/P‐2023062). Informed consent was obtained from the legal guardians of all cases included in the study.

### 2.2 Data Sources and Case Group Selection

Patient medical records were extracted via our institution's “Healthcare Big Data Research Innovation Platform” (Figure 1). To safeguard patient privacy, personally identifiable information, including names and contact details, has been anonymized.

**FIGURE 1:**
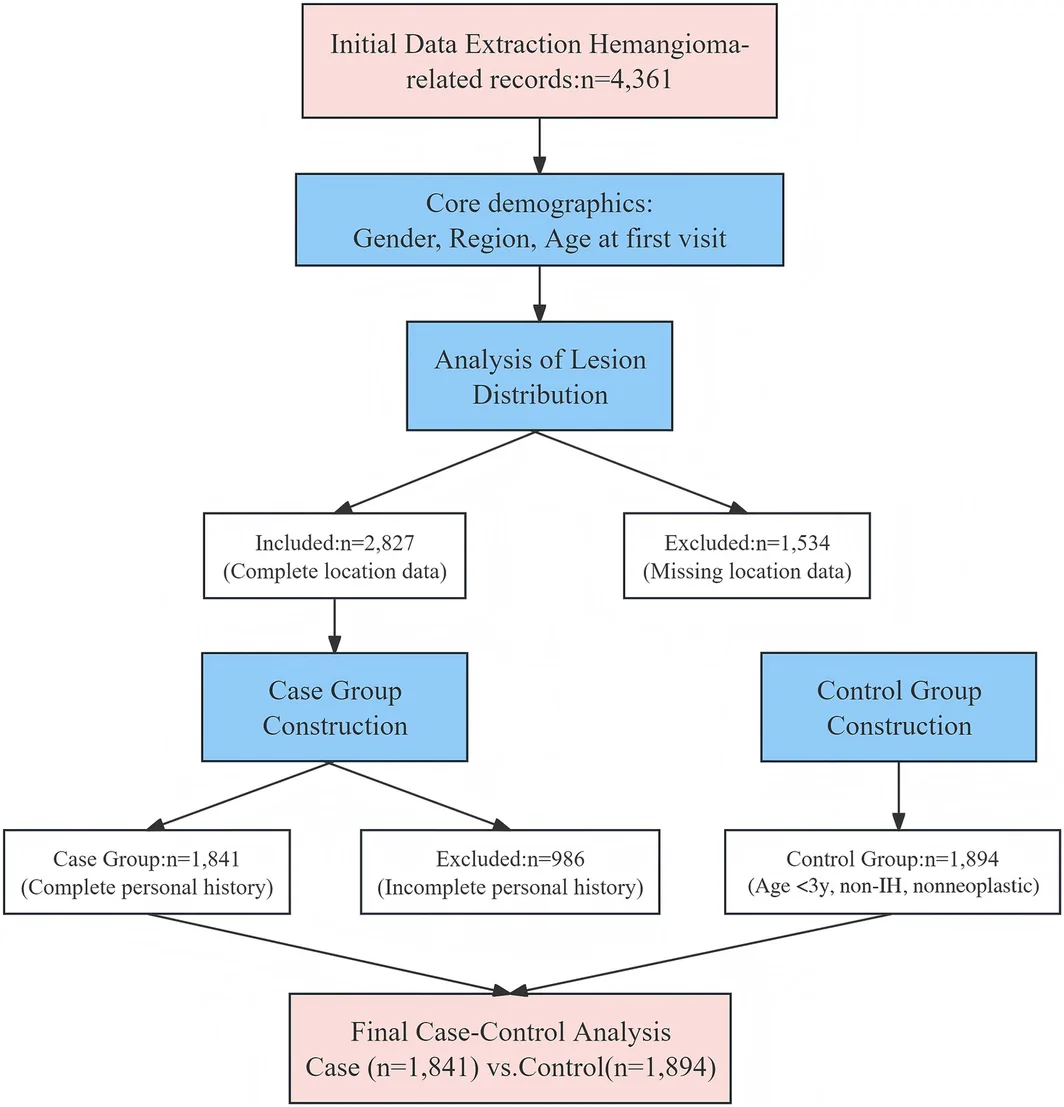
Study cohort flowchart.

The case group (IH group) selection process was as follows:

- Initial cohort: Data for all patients with concurrent hemangioma‐related consultations (*n* = 4 361) were extracted from the platform.
- Descriptive Analysis Cohort: All 4 361 patients were included in descriptive statistical analysis of core demographic characteristics (e.g., gender, region of residence, age at first consultation).
- Lesion Distribution Analysis Cohort: 1 534 patients lacking definitive lesion location information were excluded from lesion distribution analysis, resulting in 2 827 patients included.
- Case–control cohort for risk factor analysis: To ensure baseline data completeness and study reliability, an additional 986 patients with incomplete personal history records (including key information such as birth circumstances and maternal pregnancy‐related factors) were excluded. Ultimately, 1 841 IH children with complete records of key study‐required information were included in the case cohort for risk factor analysis. All children in the case cohort met the following inclusion criteria: Confirmed diagnosis of IH, age under 3 years, and absence of other congenital tumors.

### 2.3 Control Group Selection

The control group (non‐IH cohort) was selected according to strict matching principles to ensure comparability between groups. The control group comprised 1 894 pediatric patients treated concurrently within the same department. Inclusion criteria were: Age under 3 years, absence of IH, no congenitaltumors, and availability of comprehensive clinical data. The case–control ratio was approximately 1:1 to enhance statistical power.

### 2.4 Data Extraction and Variable Definitions

During the extraction of clinical and perinatal data for the risk factor analysis, specific exposure variables were strictly defined. “Breastfeeding” was defined as predominant breast milk intake during early infancy, where breast milk served as the infant's primary source of nutrition. Additionally, “Pregnancy‐related diseases” documented in the maternal history primarily referred to the occurrence of gestational hypertension and/or gestational diabetes mellitus.

### 2.5 Use of Artificial Intelligence‐Assisted Technologies

In accordance with journal guidelines, the authors declare the use of an artificial intelligence‐based large language model (Gemini) during the preparation of this manuscript.

The tool was utilized strictly to assist with English language translation, grammatical polishing, and structural refinement of the text. The AI tool was not used for data extraction, statistical analysis, or drawing primary scientific conclusions.

After using this service, the authors rigorously reviewed and edited the content, verified all scientific claims against the primary literature, and take full responsibility for the final content and integrity of the publication.

## 3 Statistical Analysis

All statistical analyses in this study were performed using R software version 4.5.1, with a statistical significance level set at α = 0.05. In descriptive analysis, categorical variables such as gender, age at initial diagnosis, geographical distribution, and lesion distribution were presented as “number of cases (percentage) [n (%)]”.

In univariate analysis, all included variables were categorical. Intergroup comparisons employed the χ^2^ test. Potential factors preliminarily associated with IH occurrence were screened through univariate analysis. Variables demonstrating statistical association (*p* < 0.05) were incorporated into a multivariate logistic regression model to identify independent determinants of IH development. Statistical significance was defined as *p* < 0.05 across all analyses.

## 4 Results

### 4.1 Current Epidemiology of Infantile Hemangioma (Table 1)

**TABLE 1:** Baseline characteristics of the subjects in our cohort.

Characteristic variable	Category	Frequency (*n*)	Percentage (%)
Gender	Female	2 846	65.3%
	Male	1 515	34.7%
Age at initial presentation	0–1 months	200	4.6%
	1–3 months	1 805	41.4%
	3–6 months	1 281	29.4%
	6–12 months	717	16.4%
	> 12 months	358	8.2%
Regional distribution	Ji Nan	1 841	42.2%
	He Ze	448	10.3%
	De Zhou	329	7.5%
	Liao Cheng	279	6.4%
	Ji Ning	227	5.2%
	Other Cities	1 237	28.4%
Lesion distribution	Head and Neck	1 415	46.7%
	Trunk	749	24.7%
	Limbs	732	24.1%
	Perineum	93	3.1%
	Visceral	41	1.4%

(1) Gender Distribution: Female patients numbered 2 846 (65.3%), while male patients totaled 1 515 (34.7%), resulting in a male‐to‐female ratio of approximately 1:1.88.

(2) Time Distribution Characteristics: The majority of initial consultations occurred between 1 and 3 months of age, accounting for 1 805 cases (41.4%). This was followed by 1 281 cases (29.4%) between 3 and 6 months, and 717 cases (16.4%) between 6 and 12 months. The proportion of patients presenting at 0 to 1 month of age and after 12 months of age was relatively low, at 4.6% and 8.2%, respectively. The peak presentation period occurred between 1 and 3 months of postnatal age, highlighting the rapid proliferative phase typically commencing in early infancy.

(3) Regional distribution characteristics: Patients predominantly originated from Ji Nan City (1 832 cases, 42.2%), followed by He Ze City (10.3%), De Zhou City (7.5%), Liao Cheng City (6.4%), and Ji Ning City (5.2%). Other cities accounted for 28.4%. This distribution exhibits a decreasing trend radiating outward from diagnostic centers, reflecting the influence of healthcare accessibility on patient referral patterns and indicating potential sampling bias relative to actual population epidemiology.

(4) Lesion Characteristics in IH: Among 2 827 patients with detailed lesion records, 3 030 lesions were documented, indicating several lesions in some individuals. Anatomical distribution showed the head and neck as the most common site (1 415 lesions, 46.7%), followed by the trunk (749 lesions, 24.7%) and the limbs (732 lesions, 24.1%). The perineum (93 lesions) and visceral involvement (41 lesions) were relatively uncommon.

### 4.2 Univariate Analysis

Univariate statistical inference conducted on the case group (*n* = 1 841) and control group (*n* = 1 894) (Table 2) revealed statistically significant differences between the two groups in the following factors: Female, pregnancy‐related diseases (including gestational hypertension and diabetes), progestogen therapy, preterm birth, low birth weight, multiple gestation, breastfeeding, and caesarean. No significant statistical association was observed between the risk of occurrence and asphyxia (*p* = 0.397), miscarriage (*p* = 0.113), parity (first pregnancy) (*p* = 0.788), or fetus number (first birth) (*p* = 0.616).

**TABLE 2:** Univariate analysis of factors influencing IH.

Influence factor	IH Group (*n* = 1 841)	Control Group (*n* = 1 894)	Statistical Value ($\chi^{2}$)	*p*
Female	1 204 (65.4%)	905 (47.8%)	89.461	< 0.001
Pregnancy‐related diseases	542 (29.4%)	240 (12.7%)	118.050	< 0.001
Progestogen therapy	227 (12.3%)	99 (5.2%)	56.111	< 0.001
Preterm birth	355 (19.3%)	201 (10.6%)	54.710	< 0.001
Low birth weight	158 (8.6%)	72 (3.8%)	36.102	< 0.001
Multiple gestation	82 (4.5%)	22 (1.1%)	39.313	< 0.001
Breastfeeding	1 232 (66.9%)	1 400 (73.9%)	21.631	< 0.001
Caesarean	867 (47.1%)	959 (50.6%)	4.472	0.034
Asphyxia	171 (9.3%)	160 (8.4%)	0.716	0.397
Miscarriage	55 (3.0%)	72 (3.8%)	2.510	0.113
Parity (first pregnancy)	878 (47.7%)	891 (47.0%)	0.072	0.788
Fetus number (first birth)	888 (48.2%)	897 (47.4%)	0.252	0.616

### 4.3 Multivariate Analysis

Variables demonstrating statistical significance in univariate analysis were selected for the multivariate logistic regression model. Results revealed that multiple gestation constituted the highest independent risk factor for IH (OR = 4.50), followed by female gender (OR = 2.72), progestogen therapy (OR = 2.25), low birth weight (OR = 2.11), preterm birth (OR = 1.65), and pregnancy‐related diseases (OR = 1.57). Breastfeeding (OR = 0.76) emerged as an independent protective factor against IH; caesarean section did not demonstrate statistical significance in the multi‐factor analysis (*p* = 0.62), suggesting it may not be an independent influencing factor for IH development. The results of the multivariate analysis are presented in Table 3 and Figure 2.

**TABLE 3:** Multivariate logistic regression analysis of factors influencing IH.

Influence factor	*β*	SE	Wald	*p*	OR (95% CI)
Female	1.002	0.113	78.133	< 0.001	2.72 (2.18, 3.40)
Multiple gestation	1.504	0.121	6.021	0.0141	4.50 (3.55, 5.70)
Progestogen therapy	0.809	0.292	7.670	0.0056	2.25 (1.27, 3.98)
Low birth weight	0.745	0.313	5.674	0.0172	2.11 (1.14, 3.89)
Preterm birth	0.504	0.247	4.163	0.0413	1.65 (1.02, 2.69)
Pregnancy‐related diseases	0.450	0.219	4.201	0.0404	1.57 (1.02, 2.41)
Breastfeeding	−0.278	0.139	3.994	0.0457	0.76 (0.58, 0.99)
Caesarean	0.056	0.114	0.241	0.6231	1.06 (0.85, 1.32)

**FIGURE 2:**
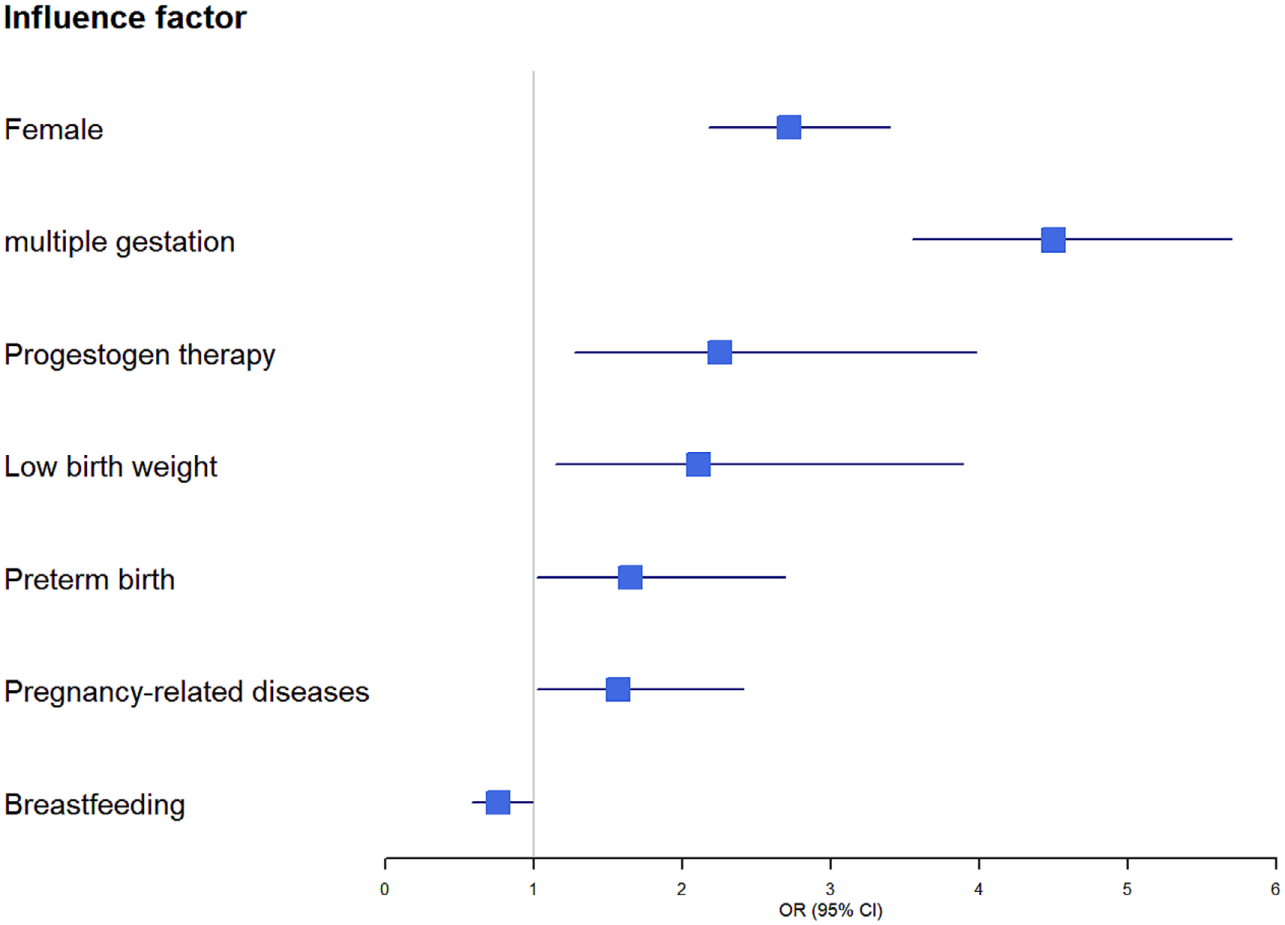
Contributing factors to IH.

## 5 Discussion

This study, based on large‐scale case–control datasets from the Chinese population, systematically elucidates the epidemiological characteristics of IH and its association with multiple perinatal factors. The findings indicate that female sex, multiple gestation, preterm birth, low birth weight, pregnancy‐related diseases (including gestational hypertension and diabetes), and progestogen therapy constitute independent risk factors for IH development, whereas breastfeeding demonstrates a clear protective effect. These discoveries not only align with the majority of global studies but also provide crucial population‐based evidence for deepening our understanding of IH pathogenesis.

Consistent with global epidemiological characteristics, our cohort confirmed a marked female predominance [6, 8, 12], a pattern potentially amplified in hospital settings by referral bias arising from parental cosmetic concerns, alongside estrogen‐driven VEGF upregulation [13, 14, 15, 16]. Furthermore, multiple gestation, prematurity, and low birth weight emerged as prominent risk factors. The underlying mechanisms of these variables are well‐documented in existing literature, primarily revolving around relative placental insufficiency, chronic intrauterine hypoxia, and the subsequent stabilization of hypoxia‐inducible factor‐1α (HIF‐1α) [17, 18, 19, 20]. This hypoxic microenvironment forcefully drives the transcription of downstream pro‐angiogenic factors like VEGF. Concurrently, prematurity and low birth weight are distinctly associated with the postnatal overactivation of the renin‐angiotensin system (RAS) [21, 22, 23]. These insights provide a crucial pathophysiological rationale for treating IH with the beta‐blocker propranolol, suggesting that its clinical efficacy may be closely linked to the established role of the RAS in IH biology, which is characterized by the expression of angiotensin‐converting enzyme (ACE) and angiotensin II receptor‐2 in endothelial progenitor cells during the proliferative phase [24]. Similarly, exogenous hormone exposure (progestogen therapy) [14] and pregnancy‐related disorders (such as pre‐eclampsia and gestational diabetes) [25, 26, 27] further contribute to this aberrant pro‐angiogenic microenvironment via placental ischemia and foetal hyperinsulinemia.

Most importantly, redirecting from these traditional risk models, our study has established that breastfeeding constitutes an independent protective factor against IH within the Chinese population. Immunomodulatory components [28], specific human milk oligosaccharides [29], and bioactive peptides [30] present in breast milk may mitigate abnormal proliferation of vascular endothelial cells by inhibiting the VEGF signaling pathway and regulating the immune microenvironment. Recent mechanistic studies have demonstrated that certain specific proteins in breast milk (such as lactoferrin) can exert direct anti‐angiogenic effects by downregulating VEGF expression and inhibiting endothelial cell migration, thereby effectively delaying or even preventing IH growth [31]. Crucially, while premonitory signs of IH frequently appear within the first two weeks of life, the transition from a microscopic precursor to a clinically significant tumor is characterized by a prolonged proliferative phase lasting up to 6 months [32, 33]. During this extended window, rapid endothelial cell mitosis is persistently driven by key angiogenic factors, notably VEGF [34]. Therefore, to effectively inhibit this prolonged “angiogenic switch” and counteract the continuous angiogenic drive, only long‐term and sustained breastfeeding throughout early infancy holds true clinical significance. Additionally, this biological plausibility is further supported by localized clinical evidence from a 2012 study demonstrating that topical breast milk application can successfully promote the healing of ulcerated IH [35].

To contextualize our findings, national epidemiological data from 2018 report an “ever breastfed” rate of 91.50% and a 6‐month exclusive breastfeeding rate of 47.90% in the general Chinese infant population [36]. The breastfeeding rate of 73.9% observed in our control group logically aligns within this national spectrum. In this context, the significantly lower rate observed in our IH cohort (66.9%) further supports the true epidemiological protective effect of predominant breast milk intake during early infancy, highlighting the public health value of targeted lactation support for high‐risk infants.

This study has several limitations. Firstly, as a single‐center, retrospective hospital case–control study, selection bias (such as Bourke bias) is unavoidable. Controls were drawn from hospitalized pediatric patients during the same period, whose exposure characteristics may differ from those of healthy infants in the community. Secondly, a significant proportion of initial cases were excluded due to missing key perinatal history data, which may have impacted the generalizability of the findings. Thirdly, despite multivariate adjustments, residual confounding may remain; since high‐risk infants (e.g., premature or low birth weight) inherently face both feeding challenges and elevated IH risks, the observed protective effect of breastfeeding might partially reflect a healthier baseline status. Future multicenter, prospective birth cohort studies employing stratified analyses are required to validate these associations and establish causality.

In summary, this study indicates that IH results from the combined effects of genetic susceptibility, specific intrauterine environmental stresses (such as multiple gestation and hypoxia), and perinatal hormone exposure. Breastfeeding, as a modifiable protective factor, holds significant public health implications. These findings provide crucial data support for epidemiological understanding, risk surveillance, and early prevention strategies for IH in China.

## 6 Conclusion

This large‐scale study elucidates the epidemiological landscape of IH in the Chinese population, identifying it as a multifactorial condition driven by female predisposition, intrauterine hypoxic stress, and perinatal hormone exposure. While factors such as prematurity, low birth weight, and progestogen therapy significantly elevate risk, breastfeeding emerges as a crucial modifiable protective factor. These findings support the hypothesis that placental insufficiency and hormonal dysregulation are pivotal in triggering abnormal vascular proliferation, underscoring the necessity for targeted surveillance of high‐risk infants and reinforcing the public health value of breastfeeding promotion for potential risk mitigation.

## Funding

The authors declare that funding was provided by Big Data Program of Jinan Municipal Health Commission (2023‐BD‐1‐10).

## Disclosure

The authors have nothing to report.

## Ethics Statement

Data extraction for this study was conducted using “the Hospital Health Big Data Research and Innovation Platform” and approved by the Institutional Review Board (IRB) of Shandong University Children's Hospital (Approval No. SDFE‐IRB/P‐2023062).

## Consent

The authors have nothing to report.

## Conflicts of Interest

The authors declare no conflicts of interest.

## Data Availability

The datasets used and analyzed during this study are available from the corresponding author on reasonable request.
